# Molecular characterization of gliotoxin-producing *Aspergillus fumigatus* in dairy cattle feed

**DOI:** 10.14202/vetworld.2023.1636-1646

**Published:** 2023-08-17

**Authors:** Hams M. A. Mohamed, Imer Haziri, AbdulRahman A. Saied, Kuldeep Dhama, Amal A. Al-Said, Suzan E. Abdou, Heba F. Kamaly, Hanan H. Abd-Elhafeez

**Affiliations:** 1 Department of Microbiology, Faculty of Veterinary Medicine, South Valley University, Qena, 83523, Egypt; 2 Department of Veterinary Medicine, Faculty of Agriculture and Veterinary, University of Prishtina “Hasan Prishtina”, 10000 Pristina, Kosovo; 3 National Food Safety Authority, Aswan Branch, Aswan 81511, Egypt; 4 Ministry of Tourism and Antiquities, Aswan Office, Aswan 81511, Egypt; 5 Division of Pathology, ICAR-Indian Veterinary Research Institute, Izatnagar-243122, Bareilly, Uttar Pradesh, India; 6 Department of Mycology, Animal Health Research Institute, Agriculture Research Center (ARC), P.O. 12618, Gizza; 7 Biochemistry Unit, Animal Health Research Institute Agriculture Research Center (ARC), P.O. 12618, Gizza; 8 Department of Forensic Medicine and Toxicology, Faculty of Veterinary Medicine, Assiut University, Egypt; 9 Department of Cell and Tissues, Faculty of Veterinary Medicine, Assiut University, Assiut 71526, Egypt

**Keywords:** *Aspergillus fumigatus*, cattle feed, gliotoxin, *gli*Z, high-performance liquid chromatography, real-time polymerase chain reaction

## Abstract

**Background and Aim:**

Several strains of *Aspergillus fumigatus* produce mycotoxins that affect the health and productivity of dairy cattle, and their presence in dairy cattle feed is a serious concern. This study aimed to determine the densities of *A. fumigatus* and gliotoxin in commercial dairy feed.

**Materials and Methods:**

More than 60 dairy feed samples were examined for fungal contamination, specifically for *A. fumigatus*, using phenotypic approaches and DNA sequencing of the *internal transcribed spacer* (*ITS*) and β-tubulin regions. Thin-layer chromatography and high-performance liquid chromatography (HPLC) were used to assess gliotoxin production in *A. fumigatus*. Real-time polymerase chain reaction (RT-PCR) was used to investigate the expression of *gli*Z, which was responsible for gliotoxin production. High-performance liquid chromatography was used to detect gliotoxin in feed samples.

**Results:**

*Aspergillus* was the most commonly identified genus (68.3%). *Aspergillus fumigatus* was isolated from 18.3% of dairy feed samples. Only four of the 11 *A. fumigatus* isolates yielded detectable gliotoxins by HPLC. In total, 7/11 (43.7%) feed samples tested had gliotoxin contamination above the threshold known to induce immunosuppressive and apoptotic effects *in vitro*. The HPLC-based classification of isolates as high, moderate, or non-producers of gliotoxin was confirmed by RT-PCR, and the evaluation of *gli*Z expression levels corroborated this classification.

**Conclusion:**

The identification of *A. fumigatus* from animal feed greatly depended on *ITS* and β-tubulin sequencing. Significant concentrations of gliotoxin were found in dairy cattle feed, and its presence may affect dairy cow productivity and health. Furthermore, workers face contamination risks when handling and storing animal feed.

## Introduction

Fungal growth is detrimental to human and animal health because it reduces the nutritional value of food and feed and produces mycotoxins and allergens [1–3]. The common thermophilic fungus *Aspergillus fumigatus* has been detected in spoiled animal feed [4–7]. Because *A. fumigatus* spores are easily dispersed in air, they are a threat to animals and humans [8]. Farmworkers who come in contact with this fungus through moldy feed are at risk of developing allergies, tremorgenic toxicosis, neurological disorders, and potentially fatal systemic diseases [9, 10]. In ruminants, mastitis is caused by *A. fumigatus*, which migrates to the lungs and supramammary lymph nodes, causing respiratory symptoms, abortions, and other clinical problems [11]. *Aspergillus fumigatus* produces many secondary metabolites, such as fumagillin, fumitoxins, fumigaclavines A and C, fumitremorgins, gliotoxins, and fumiquinazolines [12–14], in which *A. fumigatus* are extremely hazardous to human and animal health (genotoxic, carcinogenic, immunosuppressive, and proapoptotic).

Gliotoxins are hazardous in two ways. First, as redox-active poisons that contribute to reactive oxygen species formation by cycling between their reduced (dithiol) and oxidized (disulfide) states [14]; second, as mixed disulfides when they react with exposed thiol groups on proteins [15]. Their toxic effects are exacerbated by high concentrations of functional ATP molecules in target cells [16]. *In vitro* studies with mammalian cell lines have demonstrated that gliotoxins promote both apoptotic and necrotic cell death, in addition to mitochondrial toxicity [17]. Due to their cytotoxic, genotoxic, and apoptotic effects, gliotoxins are believed to be virulence factors in *A. fumigatus* [18–20]. The *A. fumigatus* genome contains 13 *gli* genes, all of which are likely involved in gliotoxin generation [21]. The binuclear transcription factor Zn2Cys6, encoded by *gli*Z, plays a crucial role in controlling the expression of genes required for gliotoxin production. Bok *et al*. [21] found that adding multiple copies of *gli*Z to the *A. fumigatus* genome increased virulence and gliotoxin production, whereas replacing *gli*Z with a marker gene (Δ*gli*Z) reduced gliotoxin production to undetectable levels and prevented other *gli* cluster genes from being expressed.

A few studies have examined *A. fumigatus* strains in the environment and animal feed, such as silages [22, 23]. Recent studies have demonstrated that cow feed contains gliotoxins. However, there is little information on the prevalence of *A. fumigatus* and its mycotoxins in other animal feeds [24, 25].

In this study, we aimed to isolate *A. fumigatus* from cow feed by estimating gliotoxin synthesis using thin-layer chromatography (TLC) and high-performance liquid chromatography (HPLC), as well as evaluate the expression of *gli*Z using a real-time polymerase chain reaction (RT-PCR).

## Materials and Methods

### Ethical approval

The study does not require ethical approval.

### Study period and location

The study was conducted from January to April 2022. Samples were collected from farms and feed stores in Qena Governorate. The samples were processed at the laboratory of the Faculty of Veterinary Medicine, South Valley University, Qena.

### Sample collection and feed composition

In total, 60 samples of feed for dairy cattle were obtained from dairy farms and feed stores in Qena Province, Egypt. The feed contained 40% corn, 20% soybean oil meal, 25% wheat bran, 5% rice polish, 5% bean hull, 2% molasses, 1% NaCl, and 2% vitamin-mineral premix. For mycological analysis, the feed samples were transported to the laboratory in clean, sterile, and dry plastic bags. The samples were stored at 4°C until use.

### Isolation and identification of mold species

Mold was isolated as described by Aşkun [26], with a few modifications. Briefly, dichloran rose Bengal chloramphenicol (DRBC) medium was used for culture, according to Samson *et al*. [27]. The samples were ground into fine powder: 10 g of each sample was mixed with 90 mL of 1% peptone water and 0.1 mL aliquots were plated on DRBC media. The DRBC plates were incubated at 25°C for 7–10 days. *Aspergillus fumigatus* colonies were subcultured on potato dextrose agar. Molds were identified based on colony shape and color. Lactophenol cotton blue staining was used to examine the form and branching of hyphae, shape of mold head, arrangement, and shape of conidia under a microscope (Olympus comp., Model [CX31RTSF], Tokyo, Japan] at 100× and 400× magnifications [28–30].

### DNA extraction

DNA was extracted according to Liu *et al*. [31]. Fungal mycelia and spores were cultivated in 50 mL of Sabouraud dextrose broth (CM0041; Oxoid, UK) for 5 days. The broth was filtered to isolate DNA. Different concentrations of mycelia and spores (3–15 mg) samples were ground using liquid nitrogen and mini-grinders (1632146; Bio-Rad, CA, USA) to extract DNA from the broth. The mixture (grounded mycelia and spores with liquid nitrogen) was combined and incubated with 500 μL of lysis buffer (containing 40 g/mL of RNase A) (Fermentas, St. Leon-Rot, Germany) for 10 min at room temperature(27°C); mixed with 150 μL of potassium acetate, and centrifuged at 8000× *g* for 5 min. A fresh tube was used to centrifuge the recovered supernatant. DNA was separated after 15 min of centrifugation at 14,000× *g* by precipitation twice with 600 μL of ice-cold isopropanol and 300 L of ice-cold ethanol (Bioline, London, UK). DNA was reconstituted in 50 μL of PCR-grade water and stored at −80°C. A nanophotometer (Implen, Schatzbogen, Munich, Germany) was used to evaluate DNA concentration and purity.

### Polymerase chain reaction amplification and sequencing of the internal transcribed spacer (ITS) region and β-tubulin gene

For uniplex PCR, 0.5 μL of extracted DNA, 1.5 mM of MgCl_2_, 0.08 mM of dNTPs (Promega, Madison, USA), 0.2 pmol/L of primers (Table-1) [32–34], and 0.02 U of GoTaq DNA polymerase (Promega) were mixed in a final volume of 25 μL. The *ITS* region was amplified using PCR under the following conditions: initial denaturation at 95°C for 5 min; 30 cycles of priming denaturation at 94°C for 30 s, annealing at 55°C for 30 s, and extension at 72°C for 1 min; final extension at 72°C for 10 min; for β-tubulin: Initial denaturation at 94°C for 5 min, followed by 35 cycles of initial denaturation at 94°C for 1 min, annealing at 68°C for 1 min, and extension at 68°C for 2 min. A 10 L sample of amplified DNA was separated on a 1.5% agarose gel in 1× TBE, stained with ethidium bromide, and analyzed under UV light. The purified PCR products were sequenced on an automated DNA sequencer (Applied Biosystems, Thermo-Fisher-Scientific Co., USA) using PRISM Big Dye Terminator V3.1 Cycle Sequencing Kit (Cat. No. 4336917; Applied Biosystems).

**Table-1:** Primers sequences used.

Gene	Primer sequences 5’- 3’	Size (bp)	Reference
*ITS*	ITS1: F-TCCGTA GGT GAA CCT GCG G ITS4: R-TCC TCC GCT TAT TGA TAT GC	290	[32]
*β-tubulin*	F- TTCCCCCGTCTCCACTTCTTCATG R- GACGAGATCGTTCATGTTGAACTC	537	[33]
*gliZ*	F- ACGACGATGAGGAATCGAAC R- TCCAGAAAAGGGAGTCGTTG	177	[34]

### Phylogenetic investigation

We analyzed forward and reverse *ITS* sequences and *β*-tubulin gene sequences from the isolates. MegAlign software was used for sequence alignment (DNASTAR, Window version 3.12e Madison, WI, USA). The identity of the amplified *ITS* and β-tubulin fragments in our isolates was verified against *A. fumigatus* reference strains registered in GenBank (https://blast.ncbi.nlm.nih.gov/). We created a phylogenetic tree based on the nucleotide sequences of *ITS* and β-tubulin in four randomly selected samples of *A. fumigatus*. To preserve a maximum of 10 trees per replicate, bootstrap support evaluations [35] were performed using 1000 repetitions (each of which had 1000 repetitions of random sequence addition), equal weighting, and tree-bisection reconnection branch transitions.

### Uniplex PCR to screen *gliZ* in *A. fumigatus* isolates

Wizard Genomic DNA Purification Kit (Promega) was used for DNA extraction as described by Liu *et al*. [31]. For PCR, a reaction volume of 25 μL of *gliZ* primer was used (Table-1). The reaction mixture contained 5 μL of 10× reaction buffer without MgCl_2_ (Fermentas, USA), 0.5 μL of 10 mM dNTPs each (Fermentas), 2.5 μL of 25 mM MgCl_2_ (Fermentas), 1 U of Taq polymerase (Fermentas), 1.5 μL of primers, and 10 ng of DNA at 4 μL. Distilled water was added to reach a final volume of 25μL. The PCR reaction for *gli*Z included 35 cycles of 94°C for 30 s, 59°C for 1 min, and 72°C for 45 s. The amplified DNA (10 μL) was analyzed on a 1.5% agarose gel with 1× TBE, stained with ethidium bromide, and visualized using a gel imaging system (Wealtec, Dolphin-View, NV, USA).

### Gliotoxin production and extraction from *A. fumigatus* isolates

*Aspergillus fumigatus* strains were cultured in autoclaved yeast extract-sucrose (YES) liquid medium. *Aspergillus fumigatus* agar plugs (5 mm in diameter), cultured on malt extract agar (MEA; CM0059B; Oxoid, UK), were incubated for 7 d at 28°C in a 250 mL Erlenmeyer flask with 100 mL of YES. The inoculated broth was filtered through a Whatman No. 1 filter paper. The filtrate was extracted by mixing it with 50 mL chloroform at 25°C. The chloroform fractions were mixed and dried in a rotary evaporator set to 60°C. The dried extracts were dissolved in 200 μL of methanol and stored at −70°C until use [36].

#### Gliotoxin extraction from cattle feed samples

Gliotoxins were quantified according to Boudra and Morgavi [37]. The samples were dried for 72 h at 48°C in a forced air oven. Distilled water (10 μL) and dichloromethane (40 mL) were added to flasks containing 10 g of samples. The samples were incubated in the solvent for 2 h at room temperature, agitated by hand for 15 min, and filtered through a filter paper (Whatman Inc., Clifton, USA). The filtrate was concentrated to 3 mL by evaporation under N_2_ flow, and the resulting dry residue was redissolved in 500 μL of distilled water and methanol (1:1, v/v) for gliotoxin identification.

#### Qualitative estimation of gliotoxins with TLC

All chemicals and gliotoxin standards were purchased from Sigma-Aldrich, USA. Gliotoxin standard (5 mg) was transported in sealed amber bottles. For TLC analysis, 1 mL of gliotoxin standard was mixed with 1 mL of 100% methanol. The mixture was stored in a deep freezer at −70°C for solidification [38]. On silica gel plates, 10 μL of chloroform extract and dissolved gliotoxin standard were spotted at ~3 cm from the bottom. Thin-layer chromatography plates were developed using a 5:4:1 mobile-phase solvent solution of toluene, ethyl acetate, and formic acid. The plates were dried using warm air and exposed to UV light at 366 nm to visualize fluorescent chemicals [35]. The isolate that produced the most amount of gliotoxin was located using the relative flow (Rf) method. The spots were located using the formula Rf value = distance moved by the spot/distance moved by the solvent, and Rf values were calculated for the sample and standard gliotoxin [39].

#### High-performance liquid chromatography-based quantification of gliotoxin

Gliotoxins were separated using a gradient solvent system at room temperature on an Inertsil octadecylsilyl silica reversed-phase column (1,504.6 mm, 5 m). Glacial acetic acid (10 mL/L) was used as solvent A and acetonitrile as solvent B. The solvent program was run as follows: 10% solvent B, increased to 50% in 30 min, 90% in 4 min, and decreased to 10% in 2 min, while maintaining a flow rate of 2 mL/min. The retention of gliotoxin was assessed after 14 min at 254 nm.

### Validation of the HPLC method

The limit of detection and quantification (LOD/LOQ) of HPLC was estimated by drawing a calibration curve around the spiking standard with the lowest concentration range, 500 ng/g (the lowest range of concentration was used). Limit of detection and LOQ were determined as described by Kortei *et al*. [40];

LOD = 3 × standard deviation/slope

LOQ = 3 × LOD

Recovery was assessed to determine the exactness and precision of the analytical technique used with the following equation:

Recovery = (mount found-mount sample)/mount of stander spike × 100%

#### Total RNA extraction and cDNA synthesis

Total RNA was extracted using a Plant/Fungi Total RNA Purification Kit (Norgen Biotek Corporation, Germany). A NanoDrop 2000 spectrophotometer (Thermo Scientific, Waltham, Massachusetts, USA) was used to measure the quality and amount of extracted RNA. cDNA was synthesized in a final volume of 20 μL using PrimeScript RT Reagent Kit from Takara Biotechnology (Dalian) Co., Ltd., China, and gDNA Eraser (perfect real-time), according to the manufacturer’s instructions. The cDNA products were stored at −20°C until use.

### Quantitative RT-PCR of *gliZ*

The expression of *gliZ*, relative to that of β-tubulin, was evaluated using Bio-Rad iQ5 Multicolor Real-Time PCR Detection System (Bio-Rad Laboratories Inc., Hercules, CA, USA), as described by Gardiner and Howlett [34]. Each reaction system of 25 μL contained 12.5 μL of SYBR Premix Ex TaqII (Tli RNase Plus, Takara, Japan) (2× conc.), 2 μL cDNA template, 1 μL of each primer (20 pmol/μL), 8 μL of DNase-free water, and 0.5 μL of ROX as a passive dye. The negative control lacked reverse transcriptase. Real-time PCR included 40 cycles of 95°C for 30 s, 55°C for 30 s, and 72°C for 1 min, followed by a final extension at 95°C for 10 min. All reactions and melting curve analyses were performed in triplicate. Real-time PCR was used to measure the expression levels of the target genes using the comparative computed tomography (CT) method. To obtain the normalized CT value, CT values for each gene were normalized against that of *β-tubulin* using the equation below.

∆CT = CT (gene of interest) - CT (housekeeping gene)

### Statistical analysis

Statistical software (statistical package for the social sciences v28, IBM, Chicago, USA) was used to analyze data. A one-way analysis of variance was used to determine control mean ± standard error and differences between means.

## Results

Sixty dairy cattle feed samples were collected and screened using mycological analysis methods. *Aspergillus* spp. were the most frequently found in 41/60 (68.3%) samples (Table-2). Compared to farm samples (63.6%), feed store samples had a higher rate of positive isolates (73.6%) (Table-2). Among the positive samples, 11 isolates (4 from farm feed and 7 from feed stores) were determined to be *A. fumigatus*. However, neither the collection source nor the feed type used in dairy cattle feed differed significantly. Therefore, all feed samples were contaminated with *A. fumigatus*, regardless of the source and type of feed.

**Table-2:** *Aspergillus fumigatus* incidence (%) at different sampling locations.

Samples source	*Aspergillus* spp. isolated (n [%])	*Aspergillus fumigatus* relative density (%)
Dairy farms (n = 30)	19 (63.3)	4 (13.3)
Feed stores (n = 30)	22 (73.3)	7 (23.3)
Total (n = 60)	41 (68.6)	11 (18.3)

**Figure-1:**
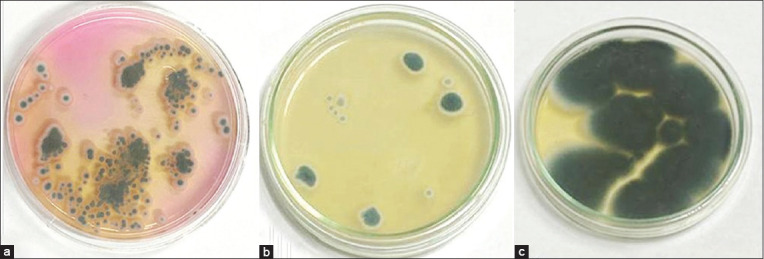
Macroscopical features of *Aspergillus fumigatus* cultivated on different media; Dichloran rose Bengal chloramphenicol (a), Potato dextrose agar (b), and Sabouraud dextrose agar (c).

**Figure-2:**
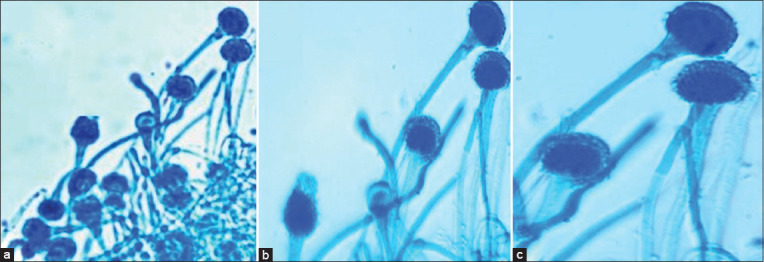
(a-c) Microscopical image of *Aspergillus fumigatus* isolates.

Four of the 11 *A. fumigatus* isolates identified phenotypically (Figures-1 and 2) were chosen randomly for molecular characterization to validate the phenotypic diagnosis.

BLAST analysis (https://blast.ncbi.nlm.nih.gov) of the amplified *ITS* and β-tubulin sequences in GenBank revealed that all the isolates belonged to a single species (*A. fumigatus*). MegAlign in Lasergene (version 7) was used to generate phylogenetic trees and multiple sequence alignments using the neighbor-joining method.

**Figure-3:**
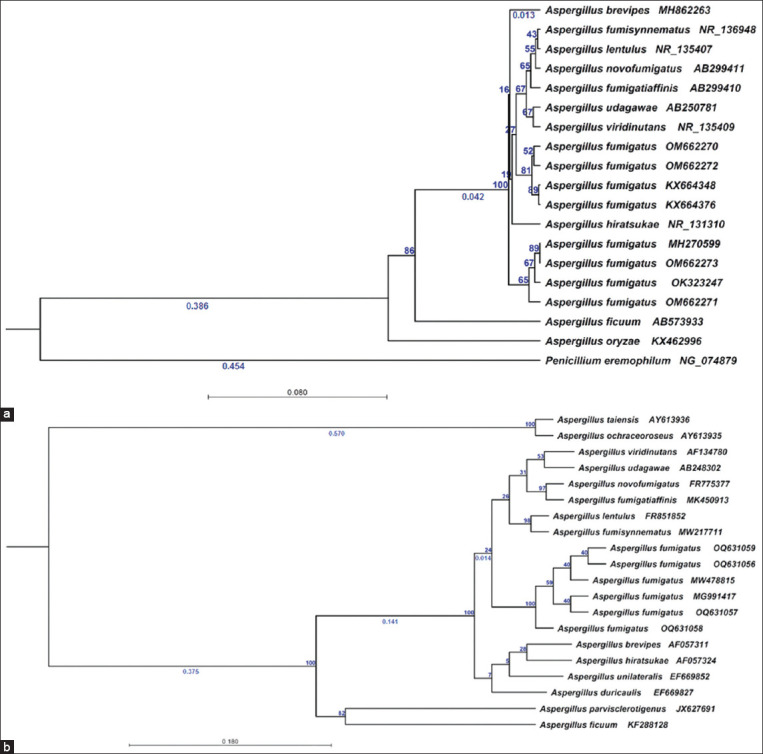
(a) The phylogenetic tree of the entire nucleotide sequence of *ITS* obtained from *Aspergillus fumigatus* strains isolated in this study compared with that of the reference strains obtained from GenBank. (b) Phylogram of *β-tubulin* showing the phylogenetic relationships among the *Aspergillus fumigatus* strains isolated in this study and strains belonging to section *Fumigati* and distinguishing the studied isolates from other *Aspergillus* spp.

Phylogenetic trees generated from the amplified *ITS* sequences of the four isolates collected from dairy cattle feed showed a high degree of similarity (Figure-3a). The degree of identity and divergence between our isolates and those published in GenBank was determined. *Aspergillus fumigatus* isolates OM662270, OM662271, OM662272, and OM662273 grouped with GenBank *A. fumigatus* reference strains MH270599, OK323247, KX664348, KX664376, and KX664343 with 100% identity.

The phylogenetic analysis of β-tubulin not only established the identity of our isolates as *A. fumigatus* but also demonstrated how they differed from other GenBank strains belonging to *Aspergillus* section *Fumigati* (Figure-3b). Clade A was the largest clade in the β-tubulin tree and contained several members of section *Fumigati*. Based on sequence divergence, these clades were classified as minor subclades. In the first subclade, *A. fumigatus* isolates OQ631056, OQ631057, OQ631058, and OQ631059 clustered with *A. fumigatus* reference strains MW478815 and MG991417, with identity ranging between 98% and 100%. Further, subclades included additional *Aspergillus* strains from section *Fumigati*, such as *Aspergillus lentulus* FR851852, *Aspergillus fumisynnematus* MW217711, and *Aspergillus novofumigatus* FR775377; these strains grouped with our isolates with an identity percentage range of 87.3%–90%.

**Figure-4:**
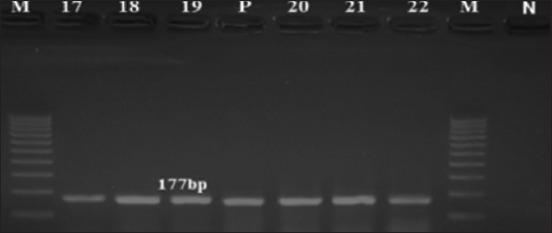
The amplification profile of *gli*Z gene in *Aspergillus fumigatus* isolates. Lane (M): DNA ladder 100 bp, lane (P): *Aspergillus fumigatus* positive control, lanes (17–22): positive isolates, and lane (N) negative control.

PCR analysis of 11 *A. fumigatus* isolates revealed that all isolates harbored *gli*Z at 177 bp (Figure-4). Thin-layer chromatography and HPLC were used to analyze gliotoxin production and determine the toxicity of the *A. fumigatus* isolates. Thin-layer chromatography analysis demonstrated that gliotoxin was produced by 3/11 (27.2%) isolates. However, HPLC analysis showed gliotoxin production by 4/11 (36.3%) isolates. Among the four positive isolates, one was classified as a high producer and three as intermediate producers. The remaining isolates were categorized as non-producers.

The natural incidence of gliotoxin (μg/g) in cattle feed was estimated using HPLC in 16 feed samples chosen randomly. Of these, 7 (43.7%) feed samples were highly contaminated with gliotoxin, with a mean amount of 1.13 ± 0.77 μg/g (Table-3) [41–43].

**Table-3:** Gliotoxin extraction levels from *Aspergillus fumigatus* isolates and dairy cattle feed samples based on HPLC.

Source of gliotoxin	Contamination frequency	Gliotoxin production level*	Range	Mean±SD
*Aspergillus fumigatus* isolates (n = 11)	4 (36.3%)	One high producer Three moderate producers	(13.6 µg/g) (0.55–1.50 µg/g)	4.14±6.326 µg/L
Feed samples (n = 16)	7 (43.7%)	High level	(0.013–2.31 µg/g)	1.13±0.77 µg/g

* The categorization of gliotoxin production levels (low/moderate/high) by *Aspergillus fumigatus* isolates follows Pena *et al*. [41], Upperman *et al*. [42], and Watanabe *et al*. [43]. SD=Standard deviation, HPLC=High-performance liquid chromatography

Validation was performed on the standard solution and feed samples. The limit of detection and LOQ values detected from the calibration curve were 0.1515 and 0.500 μg/mL, respectively. Recoveries ranged between 70.6% and 104%, with a correlation coefficient = 0.999627 (Supplementary Table-1 shows the HPLC validation parameters for gliotoxin determination.).

Traditional PCR showed that all 11 *A. fumigatus* isolates had *gli*Z. However, TLC results revealed that only three of the isolates produced gliotoxin, whereas HPLC results showed that four of the isolates produced gliotoxin (Table-4).

**Table-4:** Screening for gliotoxin production by TLC, HPLC, and PCR.

Sample ID	TLC	HPLC	PCR detection of *gliZ*
17	+	+	+
18	-	-	+
19	-	-	+
20	+	+	+
21	-	-	+
22	-	-	+
23	-	-	+
24	-	-	+
25	-	-	+
26	+	+	+
27	-	+	+
Total	3	4	11

TLC=Thin-layer chromatography, HPLC=High-performance liquid chromatography, PCR=Polymerase chain reaction

**Figure-5:**
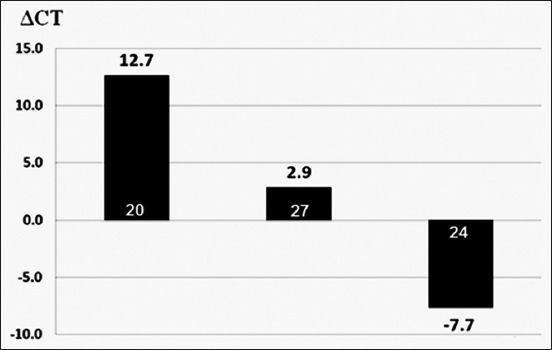
The expression level of *gliZ* in the three studied *Aspergillus fumigatus* isolates. The expression levels of the three isolates showed ΔCT values ranging from 12.7 to −7.7. A low ΔCt value indicates a low expression level, and vice versa. Based on normalized values against β-tubulin expression, samples 20, 27, and 24 showed the highest, moderate, and lowest or almost no expression levels, respectively.

**Supplementary Figure-1:**
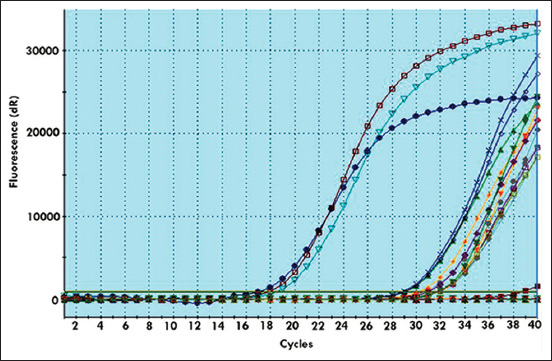
Amplification plots of *gliZ* gene expression for three isolates Based on normalized values against β-tubulin expression, samples 20, 27, and 24 showed the highest, moderate, and lowest or almost no expression levels, respectively.

Based on the HPLC results, three *A. fumigatus* isolates were chosen – one as a high producer, one as a moderate producer, and one as a non-producer – and subjected to RT-PCR to determine ΔCT values of *gli*Z expression (Figure-5). The ΔCT values ranged from 12.7 to −7.7. A low ΔCT value indicates low expression, and *vice versa*. Samples 20, 27, and 24 showed the highest, moderate, and lowest or no expression, respectively. Values were normalized against β-tubulin expression (Supplementary Figure-1 shows the amplification plots of RT-PCR system for the expression level of *gliZ* gene in the three *A. fumigatus* isolates).

## Discussion

In this study, *Aspergillus* was found to be the predominant fungal genus in dairy cattle feed. The effects of fungus in maize grain, silage, and animal feed have been documented in other studies by Adelusi *et al*. [44], Nasaruddin *et al*. [45], Cavaglieri *et al*. [46]**.** *Aspergillus fumigatus* is a frequently detected thermophilic fungus in contaminated animal feed [47, 48]. Because *A. fumigatus* spores can thrive in many environments, they are easily dispersed in air and contaminated feed and produce various mycotoxins [44]. The storage environment deters the growth of aerobic fungi and favors the growth of organisms such as *A. fumigatus* [49]. Our analysis revealed that most *A. fumigatus* isolates are present in feed stores. *Aspergillus fumigatus* was identified using both conventional and molecular methods. Although studies have supported the morphological identification of fungi to understand the evolution of morphological characteristics, traditional methods are frequently inaccurate at the species level and can be misleading d to hybridization, cryptic speciation, and convergent evolution [50–53].

Therefore, we used *ITS* sequencing and tree-building techniques to confirm phenotypic identification and evaluate phylogenetic relationships [54]. Phylogenetic analysis revealed that our isolates were 100% identical to the *A. fumigatus* reference strains deposited in GenBank and originated from farm feed and the environment. This provides information regarding the source of contamination in cattle feed [55].

BLAST analysis of GenBank sequences for the amplified regions of *β-*tubulin revealed similarities between our isolates and the *A. fumigatus* isolates registered with GenBank. Phylogenetic analysis distinguished our isolates from others belonging to section *Fumigati* and other closely related species. Several studies have used β-tubulin as a molecular marker to distinguish *A. fumigatus* from other closely related species [56, 57]. In the genomics era, molecular techniques have become indispensable tools for phenotypic analysis. Therefore, it is critical to use methods that analyze *ITS* or β-tubulin regions to differentiate between cryptic species that are believed to belong to the same complex and, thus, have almost identical morphological traits [58].

*Aspergillus fumigatus* produces an extremely toxic metabolite called gliotoxin [59]. We selected YES medium for evaluating gliotoxin production because Dheeb [60] and Hussain [61] found that for gliotoxin production, YES was ideal, probably due to its components, particularly carbohydrates (4% sucrose as a source of carbon) and yeast extract (2%).

Various approaches have been used to identify and quantify gliotoxins in domestic animal and pet feed [62]. Thin-layer chromatography or HPLC and immunological techniques have been used for gliotoxin analysis [63, 64]. We used TLC and HPLC to test gliotoxin production in *A. fumigatus* isolates. Thin-layer chromatography revealed only three isolates capable of producing gliotoxin, whereas HPLC verified four isolates as gliotoxin producers, likely due to the high precision and sensitivity of HPLC in the identification and detection of mycotoxins [65–67]. Furthermore, the HPLC results revealed that *A. fumigatus* isolates produced varying levels of gliotoxin, with some isolates producing almost no gliotoxin; this finding may be related to the degree of pathogenicity or persistence of infection, as reported by Lewis *et al*. [68]. To validate our method, several parameters were measured, most important of which was recovery. The result revealed that the recovery rate ranged from 70.6% to 104%, which is considered acceptable by Tamura *et al*. [69] who reported that good recovery rates should be >70%. Although animal feed is a major source of gliotoxin contamination, no gliotoxin limits are recommended for the diets of pets or domestic animals. *Aspergillus fumigatus* mycotoxins, such as gliotoxin, are toxic to cattle and can cause deterioration in health, protein deficiency, malnutrition, diarrhea, irritability, abnormal behavior, and in rare cases, death [70]. Gliotoxins cause enterocyte apoptosis, suppression of effective T-cell responses, and monocyte apoptosis, leading to immune system dysfunction [71]. However, studies have reported that gliotoxins have immunosuppressive and apoptotic effects at concentrations <0.01 μg/mL [72]. In our study, HPLC analysis revealed higher levels of gliotoxin (1.13 ± 0.77 μg/g) (Table-3). Pereyra *et al*. [72] reported a high incidence of gliotoxin in the feed of pigs, poultry, horses, pets, and cattle.

A cluster of *gli* genes regulates gliotoxin production in *A. fumigatus*. *gli*Z that is the most important, which is responsible for gliotoxin secretion and controls the expression of other *gli* genes [73]. According to Mead *et al*. [74], *A. fumigatus* strains carrying *gliZ* are more virulent and pathogenic than strains lacking it. PCR analysis confirmed the presence of *gli*Z in all 11 *A. fumigatus* isolates were assessed; however, only four isolates were confirmed as gliotoxin producers by HPLC. This was attributed to the loss of *gli*Z expression in some isolates. *gli*Z is the main producer of gliotoxin; however, several *gli* genes produce gliotoxin and influence *gli*Z expression [74]. Therefore, the inactivation of these genes by mutations or environmental factors may affect *gliZ* expression.

Real-time quantitative RT is a sensitive, reliable, and accurate method to identify mRNA in tissues. This method detects and monitors fluorescent signals generated during amplification, obviating the need for post-PCR processing [75]. In our investigation, the results of RT-PCR demonstrated that *gli*Z expression at the level of the three isolates confirmed the HPLC findings in classifying these isolates as high, moderate, or non-producers of gliotoxin. Real-time PCR has been recommended by several authors for measuring *gli*Z expression [76]. Nevertheless, there is limited information on *A. fumigatus* and its mycotoxins in animal feed. In cattle feed, gliotoxins can affect animal productivity and health. Furthermore, farm workers who handle improperly stored animal feed face the risk of contamination.

## Conclusion

The identification of *A. fumigatus* from animal feed was greatly dependent on *ITS* and β-tubulin sequencing. Thin-layer chromatography, HPLC, and RT-PCR analyses were used to confirm gliotoxin production by these isolates. Significant concentrations of gliotoxin were found in ready-to-feed cattle feed, and its presence may affect dairy cow productivity and health. Furthermore, workers face contamination risks when handling and storing animal feed. In Egypt, various control measures, such as using varieties resistant to fungal growth, improving drying and storage conditions, and adding nanoparticles and natural oil in feed components, were studied to inhibit mycotoxin biosynthesis and prevent the growth of mycotoxigenic fungi in animal feed. These strategies can reduce the risk of mycotoxin on human and animal health.

## Data Availability

Supplementary data can be available from Hams M.A. Mohamed upon a request.

## Authors’ Contributions

HMAM: Conceptualization and implementation of the study. HMAM and HHA: Procured the materials, conducted the experiments, laboratroy work, and drafted the manuscript. HMAM and AAS: Analysis of data and drafted the manuscript. KD, AAS, and IH: Designed the study, analysis of data, reviewing the research in terms of writing and linguistic corrections. AAA, SEA, and HFK: Interpretation of data, drafted the manuscript, and laboratory work. All authors read and approved the final manuscript.

## Appendix

**Supplementary Table**

**Supplementary Table-1:** HPLC validation parameters for gliotoxin determination

HPLC parameters	
Intercept	15,084.27
slope	1,765.71
R^2^	0.999627
Regression equation	Y = 15,084.27X + 1,765.71
LOQ	0.50
LOD	0.1515
Recovery	70.6–104
Accuracy	99.9 ± 0.311

**Supplementary Figure**

## References

[1] Haque M.A, Wang Y, Shen Z, Li X, Saleemi M.K, He C (2020). Mycotoxin contamination and control strategy in human, domestic animal and poultry:A review. Microb. Pathog.

[2] Navale V, Vamkudoth K.R, Ajmera S, Dhuri V (2021). *Aspergillus* derived mycotoxins in food and the environment:Prevalence, detection, and toxicity. Toxicol. Rep.

[3] Kumar M, Chand R, Shah K (2018). Mycotoxins and pesticides:Toxicity and applications in food and feed. In:Microbial Biotechnology.

[4] Richardson M.D, Rautemaa-Richardson R (2019). Biotic environments supporting the persistence of clinically relevant mucormycetes. J. Fungi (Basel).

[5] Müller W.A, Pasin M.V.A, Sarkis J.R, Marczak L.D.F (2021). Effect of pasteurization on *Aspergillus fumigatus* in apple juice:Analysis of the thermal and electric effects. Int. J. Food Microbiol.

[6] Haas D, Pfeifer B, Reiterich C, Partenheimer R, Reck B, Buzina W (2013). Identification and quantification of fungi and mycotoxins from Pu-erh tea. Int. J. Food Microbiol.

[7] Minooeianhaghighi M.H, Shahri A.M.M, Taghavi M (2021). Investigation of feedstuff contaminated with aflatoxigenic fungi species in the semi-arid region in Northeast of Iran. Environ. Monit. Assess.

[8] Xu J (2022). Assessing global fungal threats to humans. mLife.

[9] Hui Y. H, Smith R, Spoerke D. G (2000). Foodborne Disease Handbook:volume 3. Plant Toxicants.

[10] Fashola M.O, Ajilogba C.F, Aremu B.R, Babalola O.O (2023). Airborne fungi and mycotoxins. In:Aeromicrobiology. Elsevier, Amsterdam.

[11] Hajibemani A, Sheikhalislami H (2020). Zoonotic pathogens cause of animal abortion and fetal loss. J. Zoonotic Dis.

[12] Arastehfar A, Carvalho A, Houbraken J, Lombardi L, Garcia-Rubio R, Jenks J.D, Rivero-Menendez O, Aljohani R, Jacobsen I.D, Berman J (2021). *Aspergillus fumigatus* and aspergillosis:From basics to clinics. Stud. Mycol.

[13] Dogi C, Alonso V, Fochesato A, Poloni V, Cavaglieri L (2015). Comparison of toxicogenic and immunosuppressive capacity of *Aspergillus fumigatus* strains isolated from clinical and corn silage samples. J. Appl. Microbiol.

[14] Chmielowiec-Korzeniowska A, Trawińska B, Tymczyna L, Bis-Wencel H, Matuszewski Ł (2021). Microbial contamination of the air in livestock buildings as a threat to human and animal health-a review. Ann. Anim. Sci.

[15] Gayathri L, Akbarsha M.A, Ruckmani K (2020). *In vitro* study on aspects of molecular mechanisms underlying invasive aspergillosis caused by gliotoxin and fumagillin, alone and in combination. Sci. Rep.

[16] Downes S.G, Doyle S, Jones G.W, Owens R.A (2023). Gliotoxin and related metabolites as zinc chelators:Implications and exploitation to overcome antimicrobial resistance. Essays Biochem.

[17] Ye W, Liu T, Zhang W, Zhang W (2021). The toxic mechanism of gliotoxins and biosynthetic strategies for toxicity prevention. Int. J. Mol. Sci.

[18] Bernardo P.H, Brasch N, Chai C.L.L, Waring P (2003). A novel redox mechanism for the glutathione-dependent reversible uptake of a fungal toxin in cells. J. Biol. Chem.

[19] Nguyen V.T.T, König S, Eggert S, Endres K, Kins S (2022). The role of mycotoxins in neurodegenerative diseases:Current state of the art and future perspectives of research. Biol. Chem.

[20] Cano P, Puel O, Oswald I.P (2018). Mycotoxins:Fungal secondary metabolites with toxic properties. In:Fungi. CRC Press, Boca Raton.

[21] Bok J.W, Chung D, Balajee S.A, Marr K.A, Andes D, Nielsen K.F, Frisvad J.C, Kirby K.A, Keller N.P (2006). GliZ, a transcriptional regulator of gliotoxin biosynthesis, contributes to Aspergillus fumigatus virulence. Infect. immun.

[22] González-Jartín J.M, Ferreiroa V, Rodríguez-Cañás I, Alfonso A, Sainz M.J, Aguín O, Vieytes M.R, Gomes A, Ramos I, Botana L.M (2022). Occurrence of mycotoxins and mycotoxigenic fungi in silage from the North of Portugal at feed-out. Int. J. Food Microbiol.

[23] Dos Santos V.M, Dorner J.W, Carreira F (2003). Isolation and toxigenicity of *Aspergillus fumigatus* from moldy silage. Mycopathologia.

[24] Ulrikh E.V, Smolovskaya O.V (2021). Mycotoxins in fodder and its importance on safety of feed and the health of farm animals:A review. Online J. Anim. Feed Res.

[25] Soleiro C.A, Pena G.A, Cavaglieri L.R, Coelho I, Keller L.M, Dalcero A.M, Rosa C.A.R (2013). Typing clinical and animal environment *Aspergillus fumigatus* gliotoxin producer strains isolated from Brazil by PCR-RFLP markers. Lett. Appl. Microbiol.

[26] Aşkun T (2006). Investigation of fungal species diversity of maize kernels. J. Biol. Sci.

[27] Samson R.A, Hoekstra E.S, Frisvad J.C, Filtenborg O (2002). Introduction to Food and Airborne Fungi. Centraalbureau Voor Schimmelcultures, Utrecht, Netherlands.

[28] Pitt J.I, Hocking A.D (2009). Fungi and Food Spoilage.

[29] Domsch K.H, Gams W, Anderson T.H (2007). Compendium of Soil Fungi.

[30] Ellis D, Davis S, Alexiou H, Handke R, Bartley R (2007). Descriptions of Medical Fungi. University of Adelaide, Australia.

[31] Liu D, Coloe S, Baird R, Pedersen J (2000). Rapid mini-preparation of fungal DNA for PCR. J. Clin. Microbiol.

[32] White T.J, Bruns T, Lee S, Taylor J (1990). Amplification and direct sequencing of fungal ribosomal RNA genes for phylogenetics. PCR Protocols:A Guide to Methods and Applications.

[33] Leslie J.F, Bandyopadhyay R, Visconti A (2008). Mycotoxins:Detection Methods, Management, Public Health and Agricultural Trade. CABI, United Kingdom.

[34] Gardiner D.M, Howlett B.J (2005). Bioinformatic and expression analysis of the putative gliotoxin biosynthetic gene cluster of *Aspergillus fumigatus*. FEMS Microbiol. Lett.

[35] Felsenstein J (1985). Confidence limits on phylogenies:An approach using the bootstrap. Evolution.

[36] Belkacemi L, Barton R.C, Hopwood V, Evans E.G (1999). Determination of optimum growth conditions for gliotoxin production by *Aspergillus fumigatus* and development of a novel method for gliotoxin detection. Med. Mycol.

[37] Boudra H, Morgavi D.P (2005). Mycotoxin risk evaluation in feeds contaminated by *Aspergillus fumigatus*. Anim. Feed Sci. Technol.

[38] Kosalec I, Pepeljnjak S, Jandrlic M (2005). Influence of media and temperature on gliotoxin production in *Aspergillus fumigatus* strains. Arh. Hig. Rada. Toksikol.

[39] Lazicka K, Orzechowski S (2010). The characteristics of the chosen mycotoxins and their toxic influence on the human and animal metabolism. Nat. Sci.

[40] Kortei N.K, Annan T, Akonor P.T, Richard S.A, Annan H.A, Wiafe-Kwagyan M, Ayim-Akonor M, Akpaloo P.G (2021). Aflatoxins in randomly selected groundnuts (*Arachis hypogaea*) and its products from some local markets across Ghana:Human risk assessment and monitoring. Toxicol. Rep.

[41] Pena G.A, Pereyra C.M, Armando M.R, Chiacchiera S.M, Magnoli C.E, Orlando J.L, Dalcero A.M, Rosa C.A.R, Cavaglieri L.R (2010). *Aspergillus fumigatus* toxicity and gliotoxin levels in feedstuff for domestic animals and pets in Argentina. Lett. Appl. Microbiol.

[42] Upperman J.S, Potoka D.A, Zhang X.R, Wong K, Zamora R, Ford H.R (2003). Mechanism of intestinal-derived fungal sepsis by gliotoxin, a fungal metabolite. J. Pediatr. Surg.

[43] Watanabe A, Kuriyama T, Kamei K, Nishimura K, Miyaji M, Sekine T, Waku M (2003). Immunosuppressive substances in *Aspergillus fumigatus* culture filtrate. J. Infect. Chemother.

[44] Adelusi O.A, Gbashi S, Adebiyi J.A, Makhuvele R, Aasa A.O, Oladeji O.M, Njobeh P.B (2022). Seasonal diversity and occurrence of filamentous fungi in smallholder dairy cattle feeds and feedstuffs in South Africa. J. Fungi (Basel).

[45] Nasaruddin N, Jinap S, Samsudin N.I, Kamarulzaman N.H, Sanny M (2021). Prevalence of mycotoxigenic fungi and assessment of aflatoxin contamination:A multiple case study along the integrated corn-based poultry feed supply chain in Malaysia. J. Sci. Food Agric.

[46] Cavaglieri L.R, Pereyra M.L.G, Pereyra C.M, Magnoli C.E, Chulze S.N, Dalcero A.M (2005). Fungal and Mycotoxin Contamination of Cow Feeding Stuffs in Argentina. In:Conference on Reducing Impact of Mycotoxins in Tropical Agriculture. Congress. Ghana, Africa.

[47] Pereyra C, Alonso V, Rosa C, Chiacchiera S, Dalcero A, Cavaglieri L (2008). Gliotoxin natural incidence and toxigenicity of *Aspergillus fumigatus* isolated from corn silage and ready dairy cattle feed. World Mycotoxin J.

[48] Gordon K.E, Masotti R.E, Wadell W.R (1993). Tremorgenic encephalopathy:A role of mycotoxins in the production of CNS disease in humans?. Can. J. Neurol. Sci.

[49] Izzo L, Mikušová P, Lombardi S, Sulyok M, Ritieni A (2022). Analysis of mycotoxin and secondary metabolites in commercial and traditional Slovak cheese samples. Toxins (Basel).

[50] Kalúzová M, Kačániová M, Bíro D, Šimko M, Gálik B, Rolinec M, Juráček M (2022). The change in microbial diversity and mycotoxins concentration in corn silage after addition of silage additives. Diversity.

[51] Madelin T.M, Madelin M.F (2020). Biological analysis of fungi and associated molds. In:Bioaerosols Handbook. CRC Press, Boca Raton.

[52] Latgé J.P, Chamilos G (2019). *Aspergillus fumigatus* and aspergillosis in 2019. Clin. Microbiol. Rev.

[53] Maharachchikumbura S.S.N, Chen Y, Ariyawansa H.A, Hyde K.D, Haelewaters D, Perera R.H, Stadler M (2021). Integrative approaches for species delimitation in *Ascomycota*. Fungal Divers.

[54] Lücking R, Aime M.C, Robbertse B, Miller A.N, Ariyawansa H.A, Aoki T, Schoch C.L (2020). Unambiguous identification of fungi:Where do we stand and how accurate and precise is fungal DNA barcoding?. IMA Fungus.

[55] Ruhnke M, Behre G, Buchheidt D, Christopeit M, Hamprecht A, Heinz W, Wolf H.H (2018). Diagnosis of invasive fungal diseases in haematology and oncology:2018 update of the recommendations of the infectious diseases working party of the German society for hematology and medical oncology (AGIHO). Mycoses.

[56] Gunathilaka M.G.R.S.S, Senevirathna R.M.I.S.K, Illappereruma S.C, Keragala K.A.R.K, Hathagoda K.L.W, Bandara H.M.H.N (2023). Mucormycosis-causing fungi in humans:A meta-analysis establishing the phylogenetic relationships using internal transcribed spacer (ITS) sequences. J. Med. Microbiol.

[57] Hibbett D.S, Taylor J.W (2013). Fungal systematics:Is a new age of enlightenment at hand?. Nat. Rev. Microbiol.

[58] Tekpinar A.D, Kalmer A (2019). Utility of various molecular markers in fungal identification and phylogeny. Nova Hedwigia.

[59] Leonardi M, Salvi D, Iotti M, Rana G.L, Paz-Conde A, Pacioni G (2021). Multilocus phylogeography of the *Tuber mesentericum* complex unearths three highly divergent cryptic species. J. Fungi (Basel).

[60] Dheeb B.I, Mohammad F.I, Hadi Y.A, Abdulhameed B.A (2013). Cytotoxic effect of aflatoxin B1, gliotoxin, fumonisin B1, and zearalenone mycotoxins on HepG2 cell line *in vitro*. Int. J. Adv. Res.

[61] Hussein A.F, Sulaiman G.M, Hashim A.J (2017). Improving conditions for gliotoxin production by local isolates of *Aspergillus fumigatus*. J. Biotechnol. Res. Cent.

[62] Desoubeaux G, Cray C (2018). Animal models of aspergillosis. Comp. Med.

[63] Savelieff M.G, Pappalardo L, Azmanis P (2018). The current status of avian aspergillosis diagnoses:Veterinary practice to novel research avenues. Vet. Clin. Pathol.

[64] Puri A, Ahmad A, Panda B.P (2010). Development of an HPTLC-based diagnostic method for invasive aspergillosis. Biomed. Chromatogr.

[65] Singh J, Mehta A (2020). Rapid and sensitive detection of mycotoxins by advanced and emerging analytical methods:A review. Food Sci. Nutr.

[66] Agriopoulou S, Stamatelopoulou E, Varzakas T (2020). Advances in analysis and detection of major mycotoxins in foods. Foods.

[67] Shanakhat H, Sorrentino A, Raiola A, Romano A, Masi P, Cavella S (2018). Current methods for mycotoxins analysis and innovative strategies for their reduction in cereals:An overview. J. Sci. Food Agric.

[68] Lewis R.E, Wiederhold N.P, Lionakis M.S, Prince R.A, Kontoyiannis D.P (2005). Frequency and species distribution of gliotoxin-producing Aspergillus isolates recovered from patients at a tertiary-care cancer center. J. Clin. Microbiol.

[69] Tamura M, Uyama A, Mochizuki N (2011). Development of a multi-mycotoxin analysis in beer-based drinks by a modified QuEChERS method and ultra-high-performance liquid chromatography coupled with tandem mass spectrometry. Anal. Sci.

[70] Whitlow L.W, Hagler W.M. (2010). Mold and mycotoxin issues in dairy cattle:Effects, prevention and treatment. Adv. Dairy Technol.

[71] Kraft S, Buchenauer L, Polte T (2021). Mold, mycotoxins and a dysregulated immune system:A combination of concern?. Int. J. Mol. Sci.

[72] Pereyra C.M, Alonso V.A, Fernández-Juri M.G, González-Pereyra M.L, Chiacchiera S.M, Rosa C.A.R, Dalcero A.M, Cavaglieri L.R (2007). Survey of *Aspergillus fumigatus* and Toxic Metabolites from Feed Intended for Animal Production and Pets. In:III Latin American Congress of Food Hygienists, Porto Seguro, Brazil.

[73] Seo H, Kang S, Park Y.S, Yun C.W (2019). The role of zinc in gliotoxin biosynthesis of *Aspergillus fumigatus*. Int. J. Mol. Sci.

[74] Mead M.E, Steenwyk J.L, Silva L.P, de Castro P.A, Saeed N, Hillmann F, Rokas A (2021). An evolutionary genomic approach reveals both conserved and species-specific genetic elements related to human disease in closely related *Aspergillus* fungi. Genetics.

[75] Covey S.D (2021). An adaptable dry lab for SYBR based RT-qPCR primer design to reinforce concepts in molecular biology and nucleic acids. Biochem. Mol. Biol. Educ.

[76] Bok J.W, Chung D, Balajee S.A, Marr K.A, Andes D, Nielsen K.F, Keller N.P (2006). GliZ, a transcriptional regulator of gliotoxin biosynthesis, contributes to *Aspergillus fumigatus* virulence. Infect. Immun.

